# Examining the impact of cue similarity and fear learning on perceptual tuning

**DOI:** 10.1038/s41598-023-40166-w

**Published:** 2023-08-10

**Authors:** Jonas Zaman, Kenny Yu, Marta Andreatta, Matthias J. Wieser, Yannik Stegmann

**Affiliations:** 1 Centre for the Psychology of Learning and Experimental Psychopathology, KU Leuven, Tiensestraat 102, Box 3726, 3000 Leuven, Belgium; 2 School of Social Sciences, University of Hasselt, Hasselt, Belgium; 3 Quantitative Psychology and Individual Differences, Faculty of Psychology and Educational Sciences, KU Leuven, Tiensestraat 102, Box 3726, 3000 Leuven, Belgium; 4 Department of Psychology, Education and Child Studies, Erasmus University Rotterdam, Post Box 1738, 3000 DR Rotterdam, The Netherlands; 5 Department of Psychology (Experimental Clinical Psychology), University of Würzburg, 97070 Würzburg, Germany

**Keywords:** Psychology, Human behaviour

## Abstract

Past research on the effects of associative aversive learning on discrimination acuity has shown mixed results, including increases, decreases, and no changes in discrimination ability. An animal study found that the type of learning experience determined the direction and extent of learning-induced changes. The current preregistered web-based study aimed to translate these findings to humans. Experiment 1 (N = 245) compared changes in stimulus discrimination between simple learning (only one oriented grating cue), coarse differential conditioning (physically distinct cues), and fine differential conditioning (physically similar cues) as well as to their three respective control groups. The discrimination task consisted of a two-alternative-forced-choice task with oriented grating stimuli. During learning, a specific orientation was paired with unpleasant pictures. Our analysis using generative modeling demonstrated weak to moderate evidence that aversive learning did not alter discrimination acuity in any of the groups. In a follow-up experiment (N = 121), we replicated these findings despite successful learning trajectories in all three groups and a more detailed assessment of discrimination acuity. Contrary to prior assumptions, our findings indicate that aversive learning does not enhance perceptual discrimination, and the presence of additional safety cues does not appear to moderate this effect.

## Introduction

In the past decades, a significant body of research has accrued demonstrating that associative fear learning can alter sensory processing at various levels of the sensory stream (for a review, see^1^). These findings inspired the idea that fear learning fosters the detection and discriminability of threat-associated stimuli via amplifying neural tuning profiles to the conditioned stimulus (i.e., the stimulus predictive for an aversive event) and inhibiting responses to physically similar stimuli. Clinical reports support this notion of enhanced stimulus detection and discriminability of cues present during a traumatic event^2^. Yet, laboratory research in humans on the perceptual sequelae of associative learning has produced mixed results. Some studies have found that associative learning leads to increased discriminability, while others have found decreased or no changes in discriminability (for a review, see^3^). This suggests that any potential relationship between associative learning and stimulus discriminability is complex and warrants further investigation.

As associative learning processes are conserved across species, researchers interested in fear conditioning frequently seek converging evidence from translational research to apply findings from animal models to understand the more complex human functions^4^. An animal study conducted by Aizenberg and Geffen^5^ found that in mice, the effects of fear learning on tone discrimination could go both ways, with fear learning sharpening or widening perceptual tuning. This effect depended on the presence of another tone during learning (i.e., differential learning) and the physical similarity between the two tones. During differential conditioning, one tone (called the conditioned stimulus or CS+ [The + or—signs denote the presence or absence of US reinforcement]) was paired with an aversive outcome (foot shock, called the unconditioned stimulus or US), while another tone (CS−) is consistently predictive of the US absence. Differential conditioning only improved perceptual discrimination with physically similar CSs (CSs: 15.0 kHz and 12.75 kHz), while physically distinct CSs (CSs: 15.0 kHz and 7.5 kHz) decreased the discrimination acuity of tones around the reinforced tone (CS+). Fear learning with only the 15.0 kHz tone (i.e., simple conditioning) yielded the worst perceptual discrimination.

Human studies, however, have yielded mixed results on the relationship between aversive learning and perceptual discrimination. For instance, in line with the work of Aizenberg and Geffen^5^ is the finding that aversive learning led to an improvement in detection thresholds and perceptual discrimination using indistinguishable odors as CS+ and CS−^6–8^. Also in line is the finding that associative learning (with monetary reward and loss as USs) led to worse perceptual discrimination using distinct tones as CSs (but also visual CSs)^9–13^). However, improvements in the discrimination of low-level visual features and odors have also been reported after associative learning with distinct CSs both in the laboratory and a web-based study^14–16^. In addition to these conflicting findings, several studies did not find a consistent change (or no change) in perceptual discrimination or threshold sensitivity^17–23^. However, the multitude of methodological differences among studies (e.g., adopted discrimination task, stimulus modality, reinforcement rate, type of US) renders a direct comparison of these findings difficult.

In light of these inconsistent findings and to bridge the gap between human and animal research, this present study aims to replicate the animal study of Aizenberg and Geffen^4^ to examine further in humans the moderating effect of cue similarity on learning-induced changes in perceptual discrimination. We focused on the effect of an additional safety cue (CS−) and its physical similarity to the threatening cue (CS+) on changes in discrimination acuity due to learning. The study consisted of two experiments with between-subjects designs. Experiment 1 compared changes in stimulus discrimination between three experimental groups: simple conditioning, coarse differential (with physically distinct CSs), fine differential conditioning (with physically similar CSs), and their respective control groups. The control groups received identical stimulus presentations as their experimental counterparts (but no aversive USs) to control for the effects of mere stimulus exposure. Experiment 2 was similar, with minor methodological differences and the omission of the control groups. We hypothesized that aversive learning would lead to decreased discrimination acuity between the conditioned stimulus (CS+) and surrounding stimuli in the simple and coarse differential conditioning group, with the most pronounced deterioration in the simple conditioning group. In contrast, we expected aversive learning to improve discrimination acuity between the CS+ and surrounding stimuli in the fine differential conditioning group.

## Material and methods

### Open practices statement

We report how we determined our sample size, all data exclusions, all manipulations, and all measures in the study^24^. Both experiments and analyses were preregistered at the Open Science Framework (experiment 1: https://osf.io/hz2sa ; experiment 2: https://osf.io/7wn3g). All data, analysis codes and the experimental materials can be accessed at https://osf.io/rkbp9/.

### Participants

In Experiment 1, a sample size estimation with a conservative effect size of f = 0.15 [see supplemental information (SI) for more details] yielded a total sample size of N = 228 (n = 38 × six groups). For Experiment 2, we adjusted the sample size estimation based on the average weighted effect size of Experiment 1 and Stegmann et al.^15^, yielding a total sample size of N = 117 (n = 39 × three groups) (see SI). Data were collected online via Pavlovia, with participants recruited via Prolific (For Experiment 1, a part of the participants was recruited locally using the University of Würzburg SONA Systems for Experiment 1 [N = 36]). Data collection lasted from December 2021 to March 2022 for experiment 1; experiment 2 was conducted in August 2022. Inclusion criteria were: age > 18 years, fluent understanding of English, normal or corrected to normal vision, and an approval rating > 80 (Only for the Prolific sample). Exclusion criteria were: Missing data on either of the discrimination phases > 10%, accuracy on the memory task < 70%, and participants who indicated they did not respond seriously during the task (self-report at the end of the experiment). As group allocation was random, data collection continued until the targeted 38 per group was reached. This led to the following final sample sizes per group: simple conditioning (SC) = 40, SC_control_ = 39, coarse differential conditioning (coarse DC) = 40, coarse DC_control_ = 38, fine differential conditioning (fine DC) = 39, fine DC _control_ = 49 in Experiment 1. For Experiment 2, the same inclusion and exclusion criteria were used, although with slightly adjusted cut-off scores: (i) the prolific approval rating was set to 90% instead of 80%, (ii) and > 15% instead of 10% of missing data on either the discrimination phases or the acquisition phase led to exclusion. The final sample sizes per group for Experiment 2 were SC = 41, coarse DC = 41, fine DC = 39. See SI for the demographics of the sample. The ethics committee of the Department of Psychology at the University of Würzburg approved all experimental procedures (approval code: GZEK 2020-43). Procedures were in agreement with the Declaration of Helsinki (Version 2008). All participants provided informed consent online. They received monetary compensation (6£, + 10£ per hour). The participants recruited via the Wurzburg SONA systems entered a lottery for one out of four 30€ Amazon coupons as compensation).

### Stimuli

Circular black-and-white sinusoidal grating stimuli (10 Hz spatial frequency) filtered with a Gaussian envelope (i.e., Gabor patch) with maximum contrast of 100% at the center served as conditioned stimuli and were used for the visual discrimination task. The Gabor patch with an orientation of 45° relative to the vertical axis served as the CS+ in all groups. Depending on the group, a different orientation was used as CS−. An orientation of 10° served as CS− in the coarse DC groups, while an orientation of 40° served as CS− in the fine DC groups. Given our similar design, these differences were based on the two-alternative forced choice discrimination data from Stegmann et al.^15^ to ensure that the selected CS-'s were easy and very difficult to discriminate from the CS+ .

### Aversive pictures

Identical to Stegmann et al.^15^, aversive pictures (pictures I26, I209, I208, I714, I276, I855, I210, I496, I287, and I440 from the Open Affective Standardized Image Set (OASIS)^25^) served as unconditioned stimuli (US).

### Protocol

**Figure 1:**
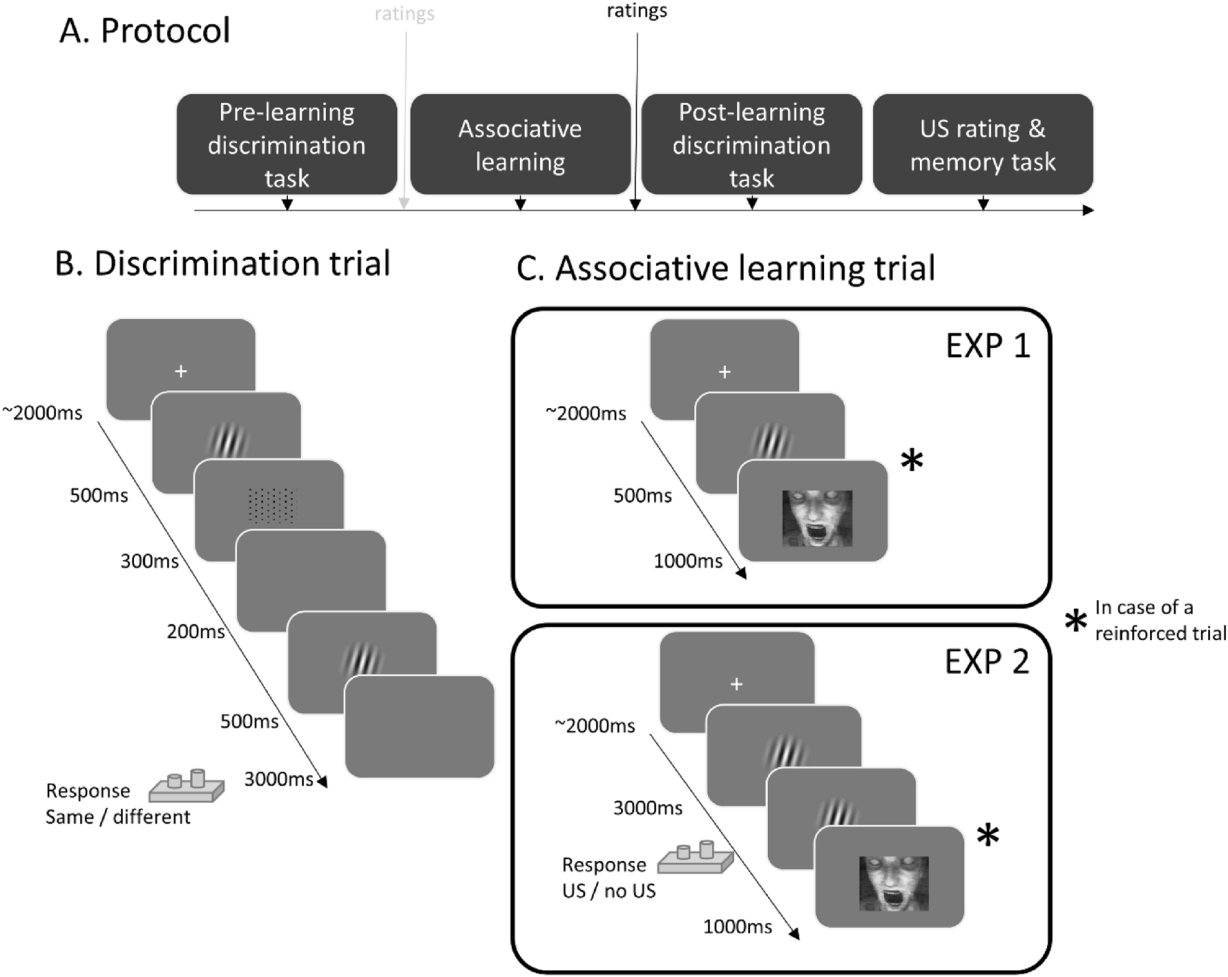
(**A**) Overview of the experimental protocol. The pre-learning threat assessment was only in Experiment 1 (grey). (**B**) Trial structure and timing of the discrimination task. (**C**) Trial structure and timing of the associative learning task in Experiment 1 and Experiment 2. Adapted from Stegmann et al.^15^ with permission.

Initially, participants were redirected to SoSciSurvey to fill in some demographic (age and gender) and the State Trait Anxiety Inventory (STAI-T) which assesses state anxiety levels^26^. The actual experimental procedure then consists of a pre and post-learning discrimination task, a classical conditioning protocol, and a US memory task (Fig. 1A). In the end, all participants indicated whether they responded seriously during the experiment or not (with the instruction that their response would not affect their compensation).

#### Discrimination task

The discrimination task comprised 160 (or 168 trials in Experiment 2) trials, each starting with a 1500–2500 ms presentation of a centrally presented fixation cross before the first Gabor patch was presented for 500 ms. This stimulus was masked by a scrambled stimulus (300 ms) and followed by a blank screen (200 ms) before the second Gabor patch appeared for 500 ms (Fig. 1B). On every trial, participants had maximally 3 s to respond whether the presented stimulus pair was identical or different via button presses. Else this was coded as a missing response (1% of the trials in Experiment 1 and Experiment 2). One of the Gabor patches always was the orientation (45°) used as CS+ (called target stimulus) and was equally likely to be presented as the first or second stimulus. The task was repeated twice, once before and once after fear conditioning. In Experiment 1, the number of presentations per orientation (range of orientations: from 15° till 75° in steps of 1°) followed a Gaussian distribution (identical to Stegmann et al.^15^). Therefore, Gabor patches near the CS+ orientation were more often presented than distinct Gabor patches (see Fig. 4). In Experiment 2, a uniform distribution was used with a more restricted range (21 stimuli, range: 35°–55°) with each stimulus presented eight times to have a more detailed measurement. The presentation order was randomly shuffled for each participant and time point. Participants did not receive feedback except from 20 extra practice trials at the beginning of the experiment, which we did not include in the analysis.

#### Fear conditioning

Two groups underwent a differential fear conditioning protocol (coarse DC and fine DC) consisting of 40 CS+ presentations and 40 CS− presentations (80 trials in total), with the CS+ trials always followed by the US (100% reinforcement rate) (Fig. 1C). The CS− was not followed by the US. In the simple conditioning group (SC), only CS+ (40 trials, again 100% reinforced by the US) but no CS− trials were presented. Each trial started with a 1500–2500 ms fixation cross, followed by the presentation of a CS Gabor patch (500 ms). If the trial was reinforced, an aversive picture was then presented for 1000 ms. Only five of the ten aversive pictures served as US in all three conditioning groups. The remaining five pictures were used for the US memory task at the end of the experiment. The choice of US was counter-balanced among participants. In the three control groups in Experiment 1 (coarse DC_control_, fine DC_control_, and SC_control_), the same number of CSs were presented as their experimental counterparts but no USs. In Experiment 2, we added two changes to the protocol: (i) the assessment of US predictions via button presses (coded as 1 = aversive picture, 0 = no aversive picture) on every trial during the conditioning phase, and (ii) that the presentation duration of the CSs was increased to 3 s. If participants responded slower than 3 s, this was registered as a missing response. These changes were implemented to assess learning trajectories throughout the conditioning phase. After the fear conditioning phase, participants rated the following range of Gabor patches (10°, 20°, 30°, 40°, 45°, 50°, 60°, 70°, 80°) on US expectancy (‘How much do you expect the presence of a picture after this stimulus?’*,* 0 = not at all; 100 = very likely). In Experiment 1, the same range of stimuli was also rated on subjective threat before and after fear conditioning.

#### US rating and memory task

At the end of the experiment—as an attention check—participants in the conditioning groups were presented with the five aversive pictures used as US, in addition to the five novel aversive pictures. Participants had to indicate (yes/no) whether the picture was presented during the conditioning session. Correct answers were summed, resulting in a memory score with a range from 0 to 10. To ensure that participants perceived the pictures as aversive, ratings for subjective arousal (“As how arousing do you perceive this picture?”, 0 = low arousing, 100 = very arousing) and valence (“As how pleasant/unpleasant do you perceive this picture?”, 0 = very pleasant, 50 = neutral, 100 = very unpleasant) were obtained using visual analog scales after each aversive picture presentation (mean arousal = 52.23 (SD = 37.51), mean valence = 82.86 (SD = 21.15), see SI for a visualization of the ratings per US).

### Analysis

All analyses were performed in R 4.1 (2020-06-06;^27^). In order to ensure clarity and comparability, we adopted the same analysis strategy for both experiments, deviating from the preregistered rmANOVA’s for Experiment 1. We used generalized linear mixed models (GLMMs) to analyze US expectancy ratings (using the lme4 package v1.1.23;^28^, which are more flexible and powerful approaches capable of dealing with continuous and binary outcomes compared to rmANOVAs^29^. US expectancy ratings were analyzed with the following linear mixed models. In Experiment 1, the within-subject predictor was Stimulus (categorical predictor: nine levels), and the between-subjects predictors were Condition (categorical predictor: simple, fine, coarse) and US (categorical predictor: US, no US). All main and interaction effects were included. In Experiment 2, the between-subjects predictor US was omitted. Based on the estimated marginal means of these models (using the EMMEANS package;^30^, we assessed the symmetry around the CS+ in the US expectancy gradient for each group using custom contrasts (coded as: ¼, ¼, ¼, ¼, 0, −¼, −¼, −¼, −¼). A lack of symmetry around the CS+ (called an area shift) would be reflective of differential learning as responding to stimuli on the non-CS− side of the CS+ tends to be higher than to equidistant stimuli on the CS− side after differential conditioning^31,32^. This phenomenon is thought to emerge due to the occurrence of both excitatory (US prediction) and inhibitory (US absence) learning in differential conditioning paradigms. However, more recent research has suggested a potential role of biased perceptual or memory mechanisms underlying this phenomenon^33^. Finally, differences between the CSs were investigated using pairwise comparisons (using the rstatix package). The result of the threat ratings from Experiment 1 can be found in the SI.

The trial-by-trial US predictions from Experiment 2 were first analyzed separately per group with a logistic GLMM that comprised Repetition (scale predictor: 1–40) and Stimulus (categorical predictor: CS+ , CS−) and their interaction as within-subject predictors. For the SC group, as only CS+ trials were presented, the predictor Stimulus and its interaction were excluded from the model. In the second step, group differences in CS+ trajectories were assessed by running a GLMM (only including CS+ trials) with Repetition (scale predictor: 1–40) as a within-subject predictor and Group (categorical predictor: coarse DC, fine DC, SC) as between-subject factor and their interaction. Lastly, differences in US predictions for the CS- trials were compared between the differential conditioning groups in a model with Repetition (scale predictor: 1–40) as a within-subject predictor and Group (categorical predictor: coarse DC, fine DC) as a between-subject factor and their interaction. The random part of the models comprised a subject-dependent intercept (and a subject-dependent effect of Stimulus when included). The logistic regression expresses the logarithm of the odds (i.e., the ratio of the probability of expecting the US compared to the probability of expecting its absence) as a linear relationship with the predictor variables. In these models, the estimated beta for the intercept is the log odds that expresses the probability of expecting the US relative to the probability of expecting the US absence (for a reference category). In our model, the CS+ was the reference category. Thus, values lower than 0, equal to 0, or greater than 0 indicates whether the probability of expecting the US was lower, equal, or higher than the probability of expecting the US absence. Beta estimates for the other predictors are log odds ratios that convey to what extent the odds change if the stimulus is a CS− compared to a CS+ .

**Figure 2:**
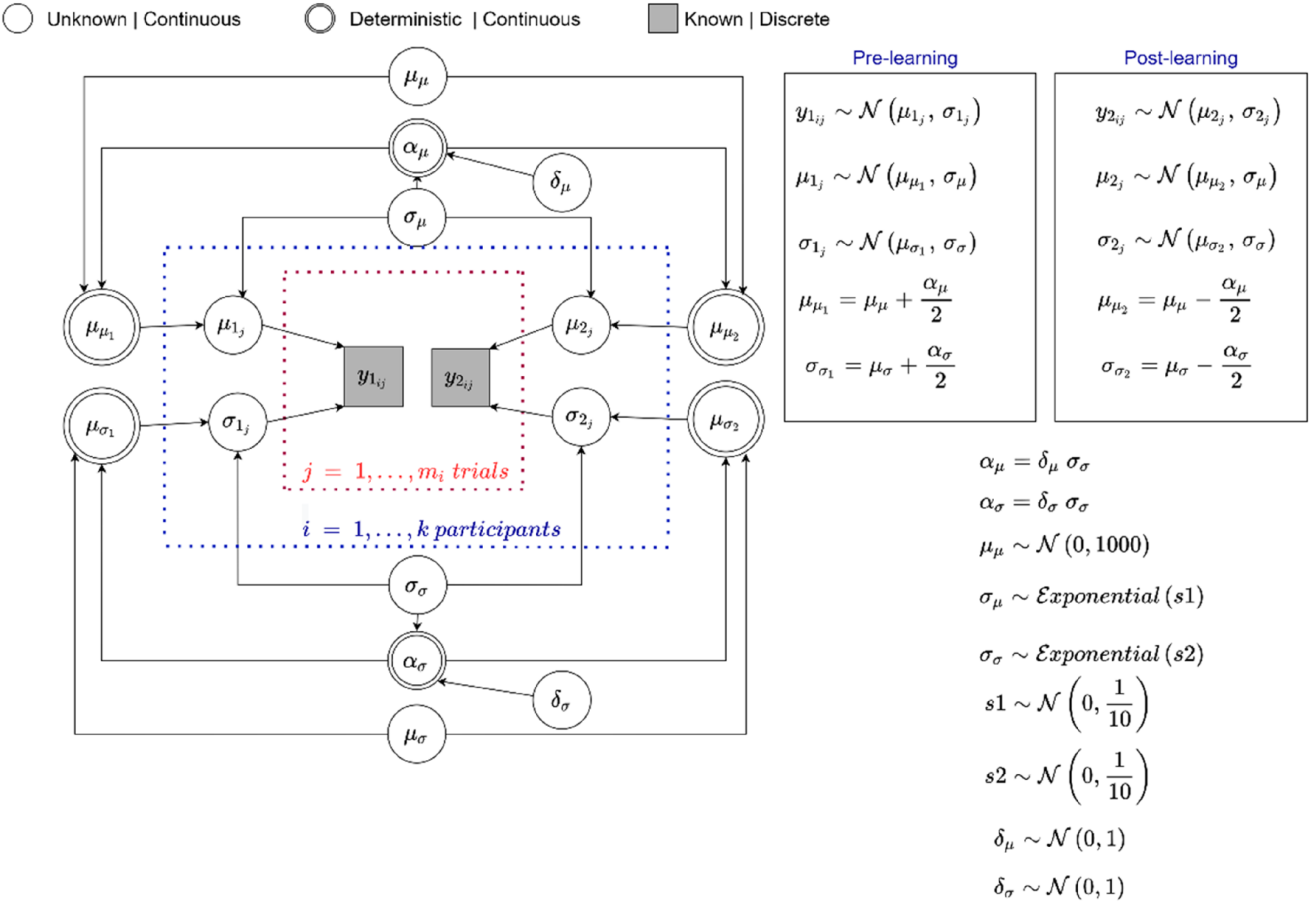
The Directed Acyclic Graph of the model for the 2AFC discrimination data.

Deviating from our preregistration, we fitted normal distributions to individuals' discrimination data in a Bayesian framework. By employing a hierarchical structure in the model, we could account for uncertainty at both the group and individual levels. Figure 2 presents a graphical representation of our model, which uses a Directed Acyclic Graph^34^ to show the connections between variables. The relevant data for the two normal distributions in the center of Fig. 2 are the responses during both the pre and post-learning discrimination task, denoted as ${\text{y}}_{{1_{ij} }}$ and ${\text{y}}_{{2_{ij} }}$ (where i = 1,…,n refers to the participant and j = 1,…,$m_{i}$ to the trial). The means ($\mu_{1}$ and $\mu_{2}$) and variances ($\sigma_{1}$ and $\sigma_{2}$) for both distributions are determined at the individual level and are further refined by group-level parameters. With the group-level parameters, we investigated to what extent the discrimination ability differs between pre-learning and post-learning conditions. We used Bayes Factors (BFs) to determine whether the standardized differences in group means and standard deviations ($\delta_{\mu }$ and $\delta_{\sigma }$) between pre and post-learning conditions were significantly different from 0, indicating a change in discriminability after learning. As a rule of thumb, BF > 10 is considered strong evidence that the standardized difference differed from 0, 3 < BF < 10 = moderate evidence, and 1 < BF < 3 = weak evidence, while BF < 1/10, 1/10 < BF < 1/3 and 1/3 < BF < 1 indicated strong, moderate or weak evidence for no difference^35^. Additionally, by comparing the difference in the posterior values of individual variance parameters between the two conditions ($\sigma_{1}$-$\sigma_{2}$), we were able to investigate how learning impacted individuals’ discriminability and the degree to which it did so. For correlations between these posterior mean parameter estimates and generalization tendencies based on US expectancy ratings, see the SI.

In all datasets, we performed parameter estimation using Markov Chain Monte Carlo (MCMC) with the Gibbs sampling method^36^ through JAGS^37^ in R (using the R2jags package). We ran three MCMC chains per model, with 30,000 iterations, discarding the first 500 iterations (i.e., burn-in). We thinned the samples by a factor of 2 for each chain (i.e., only retaining every 2^nd^ sample), resulting in 14,750 samples for each parameter. We computed the R-hat (Gelman-Rubin) statistic to assess convergence based on the three sample chains. All R-hats were below 1.01, indicating that MCMC convergence was reached. We also visually inspected the chains and confirmed that they all resembled “a fat hairy caterpillar” without any unusual pattern, indicating good convergence.

## Results

### US expectancy ratings

**Figure 3:**
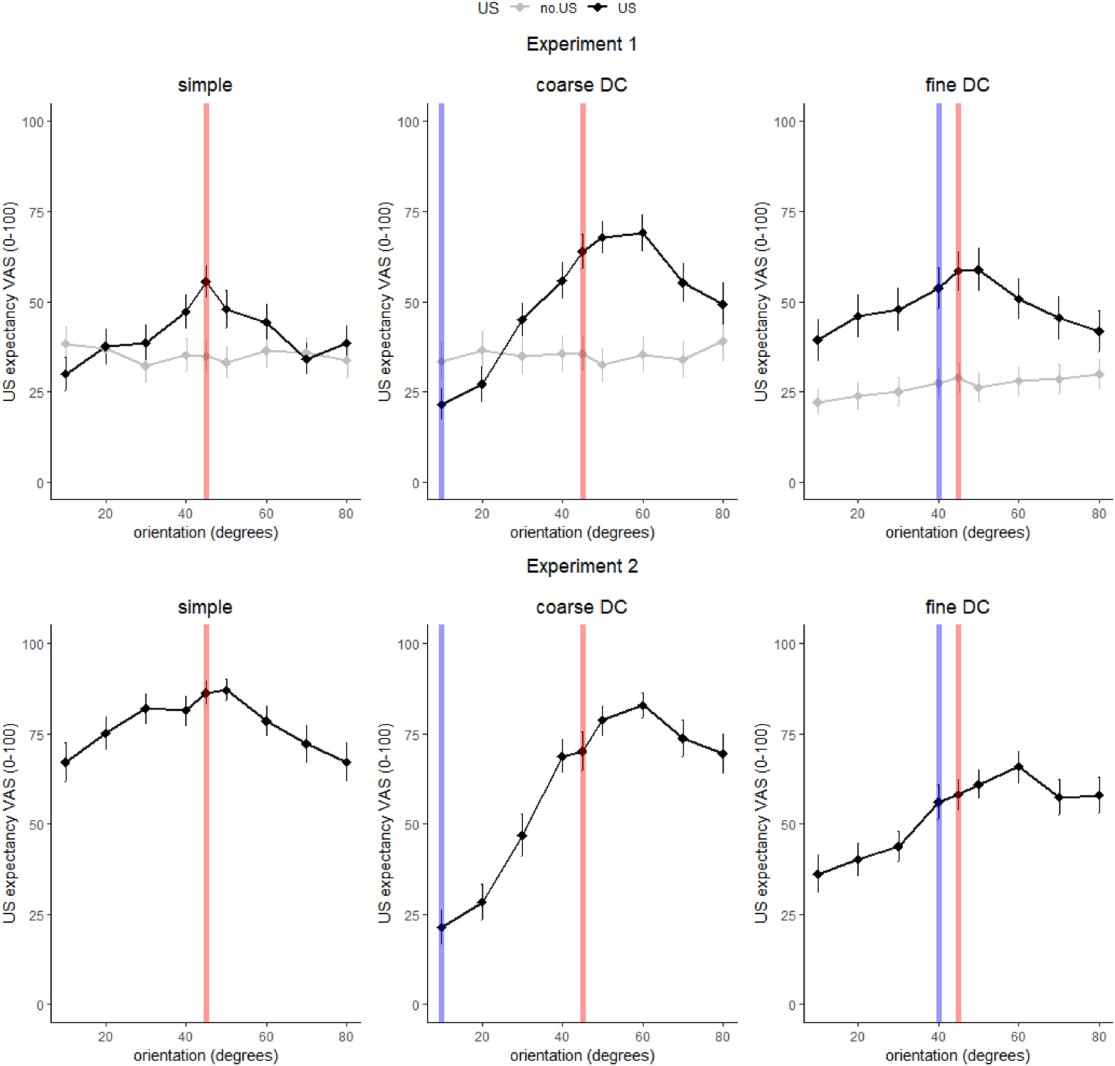
Averaged post-learning US expectancies and standard errors for Experiment 1 (top panel) and Experiment 2 (bottom panel). The grey lines in the top panel are the control conditions that received an equal amount of CSs but no USs. The red (CS+) and blue (CS−) lines indicate the position of the group's CSs on the test range.

In Experiment 1, different US expectancy gradients were found between groups across the test dimension [Condition × US × Orientation: *F*(16,1912) = 3.78, *p* < .001 $\eta_{p}^{2}$ = .03, 95% CI (0.01, 0.04)], with no differences in gradients between the control groups [US = no.US: Condition × Orientation: *F*(16,984) = 1.14, *p* = .310 $\eta_{p}^{2}$ = .02, 95% CI (0.00–0.02)] and distinct patterns in the experimental groups [US = US: Condition × Orientation: *F*(16,928) = 3.87, *p* < .001, $\eta_{p}^{2}$ = .06, 95% CI (0.02, 0.08)] (Fig. 3). Alike, we found in Experiment 2 different US expectancy gradients across the test range between the experimental groups [Condition × Orientation: *F*(16,944) = 7.33, *p* < .001, $\eta_{p}^{2}$ = 0.11, 95% CI (0.06, 0.17)] (Fig. 3). For the full model output see the SI. Next, we tested for symmetry around the CS+ in each group using custom contrasts on the model’s estimated means. The US expectancy gradient was symmetrical in the SC groups in both Experiment 1 [*t*(928) = 1.10, *p* = .270, $\eta_{p}^{2}$ = 0.00, 95% CI (0.00,0.01)] and Experiment 2 [*t*(944) = 0.05, *p* = .963, $\eta_{p}^{2}$ = 0.00, 95% CI (0.00,0.00)] and asymmetrical in the coarse DC groups in both Experiments with lower US expectancy ratings towards the CS- orientation [exp1: *t*(928) = 8.82, *p* < .001, $\eta_{p}^{2}$ = 0.08, 95% CI (0.05,0.11); exp2: *t*(944) = 12.666, *p* < .001, $\eta_{p}^{2}$ = 0.15, 95% CI (0.11,0.19)]. The pattern in the fine DC groups differed between Experiments with a symmetrical gradient in Experiment 1 [*t*(928) = 0.97, *p* = .331, $\eta_{p}^{2}$ = 0.00, 95% CI (0.00,0.01)] but not in Experiment 2 [*t*(944) = 5.850, *p* < .001, $\eta_{p}^{2}$ = 0.05, 95% CI (0.02,0.06)]. Finally, we tested for differences in US expectancy ratings between the CS+ and CS− in each group using pairwise comparisons (see Table 1). Surprisingly, no difference between the CS+ and the CS− in the fine DC groups (45° vs. 40°) was found in either Experiment 1 or 2, while ratings to these two proximal stimuli did differ in the other experimental groups in Experiment 1 but not in Experiment 2.

**Table 1:** Pairwise comparisons between the CSs per group.

	45° versus 40°	45° versus 10°
Experiment 1		
Simple	*t*(38) = 2.11, *p* = .042*	*t*(38) = 5.47, *p* < .001***
Fine DC	*t*(39) = 0.78, *p* = .435	*t*(39) = 3.10, *p* = .004**
Coarse DC	*t*(39) = 2.27, *p* = .029*	*t*(39) = 6.70, *p* < .001***
Experiment 2		
Simple	*t*(40) = 1.31, *p* = .197	*t*(40) = 3.34,* p* = .002**
Fine DC	*t*(38) = 0.60, *p* = .554	*t*(38) = 3.96, *p* < .001***
Coarse DC	*t*(40) = 0.21, *p* = .834	*t*(40) = 6.59, *p* < .001***

As the correction for multiple testing using the Holm procedure did not alter the interpretation of our findings, we only reported uncorrected *p*-values. * = *p* ≤ .05, ** = *p *≤ .01, *** *p* ≤ .001

### US predictions

**Figure 4:**
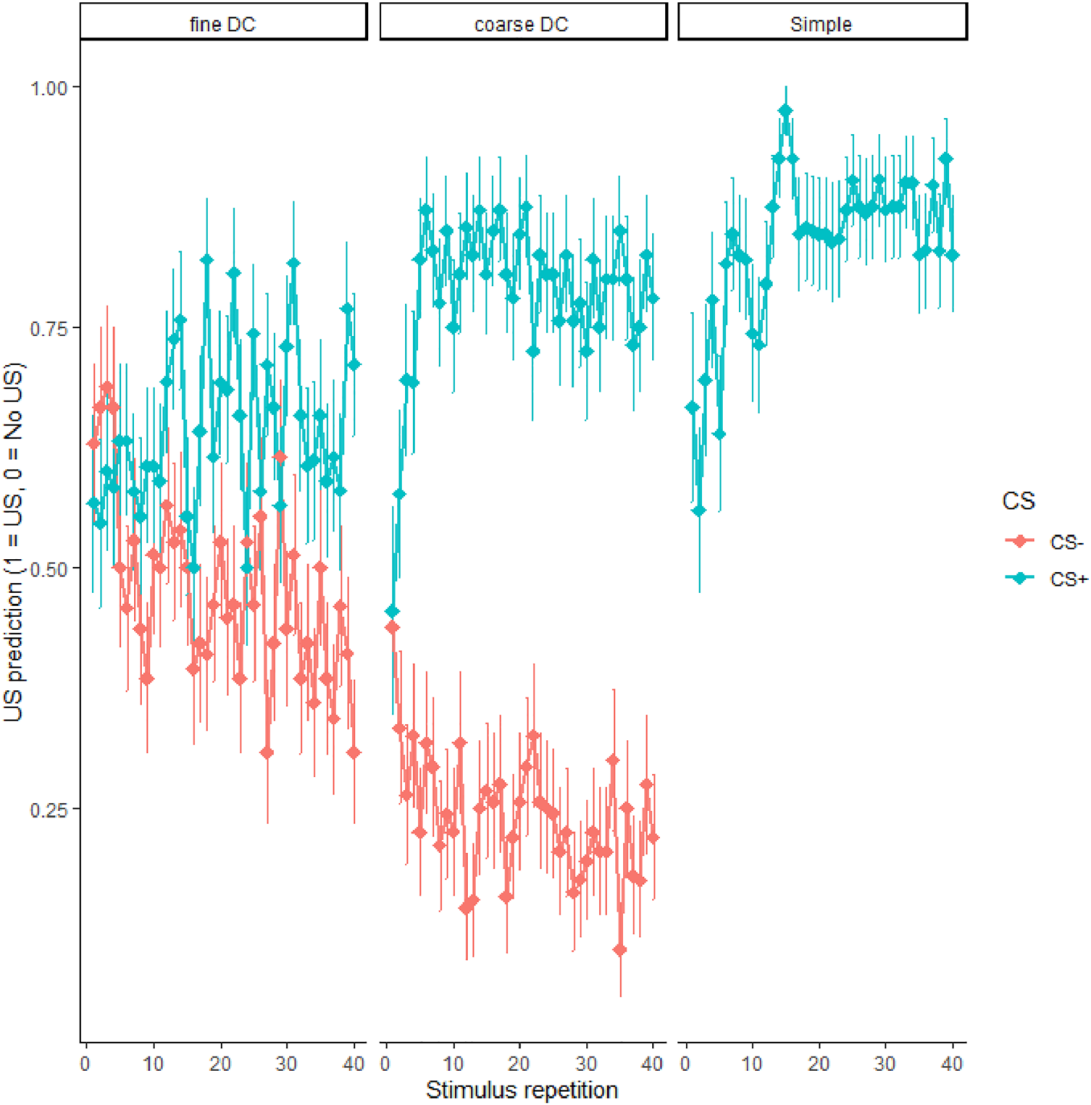
Average US predictions across fear conditioning trials in Experiment 2. Error bars denote standard errors of the mean.

In Experiment 2, we assessed US predictions on every trial as an additional measure to investigate whether learning was successful in all three conditions. Despite large interindividual differences, on average each group picked up the association between the CS(s) and the US (or it’s absence) (Fig. 4). US predictions were significantly more likely than no US predictions for CS+ trials in all three groups [SC: *β*_*intercept*_ = 1.486, *SE* = .230, *z* = 6.460, *p* < .001; fine DC: *β*_*intercept*_ = 0.488, *SE* = .177, *z* = 2.755, *p* = .006; coarse DC: *β*_*intercept*_ = 1.836, *SE* = .269, *z* = 6.833, *p* < .001] but only significantly increased across CS+ repetitions in the SC group [SC: *β*_*repetition*_ = 0.044, *SE* = .007, *z* = 6.819, *p* < .001; fine DC: *β*_*repetition*_ = 0.008, *SE* = .004, *z* = 1.845, *p* = .065; coarse DC: *β*_*repetition*_ = 0.012, *SE* = .006, *z* = 1.948, *p* = .052]. For CS− trials, in the fine DC group, predictions were not different from CS+ trials at the start [*β*_*CS-*_ = − 0.186, *SE* = .260, *z* = − 0.716, *p* = .474] but evolved differently throughout the experiment as no-US predictions became more likely [*β*_*CS-:repetition*_ = − 0.032, *SE* = .007, *z* = − 4.947, *p* < .001]. While for the coarse DC group, no-US responses were more likely during CS-trials from the start [*β*_*CS-*_ = − 3.478, *SE* = .543, *z* = − 6.404, *p* < .001] and occurred more often with CS− repetitions [*β*_*CS-:repetition*_ = − 0.043, *SE* = .009, *z* = − 4.795, *p* < .001]. Group comparisons were in line with the results from the groupwise analysis and revealed (1) more US predictions during CS+ trials in the SC and coarse DC groups compared to the fine DC [*β*_*delta*_ = 0.960, *SE* = .308, *z* = 3.120, *p* = .002];* β*_*delta*_ = 1.280, *SE* = .322, *z* = 3.972, *p* < .001]] with no difference between the former [*β*_*delta*_ = 0.320, *SE* = .327, *z* = 0.980, *p* = .327]); (2) stronger increases in US predictions across CS+ repetitions for the SC compared to both DF groups [*β*_*delta*_ = − 0.032, *SE* = .009, *z* = − 3.610, *p* < .001; *β*_*delta*_ = − 0.035, *SE* = .008, *z* = − 4.461, *p* < .001] with no difference between the latter [*β*_*delta*_ = 0.001, *SE* = .008, *z* = 0.428, *p* = .669]; (3) for CS- trials, substantially greater no-US predictions in the coarse DC compared to the fine DC group[*β*_*delta*_ = − 1.832, *SE* = .361, *z* = − 5.075, *p* < .001]; and (4) no differential increase in no-US predictions across CS− repetitions[*β*_*delta*_ = − 0.005, *SE* = .008, *z* = − 0.694, *p* = .488].

### Discrimination

**Figure 5:**
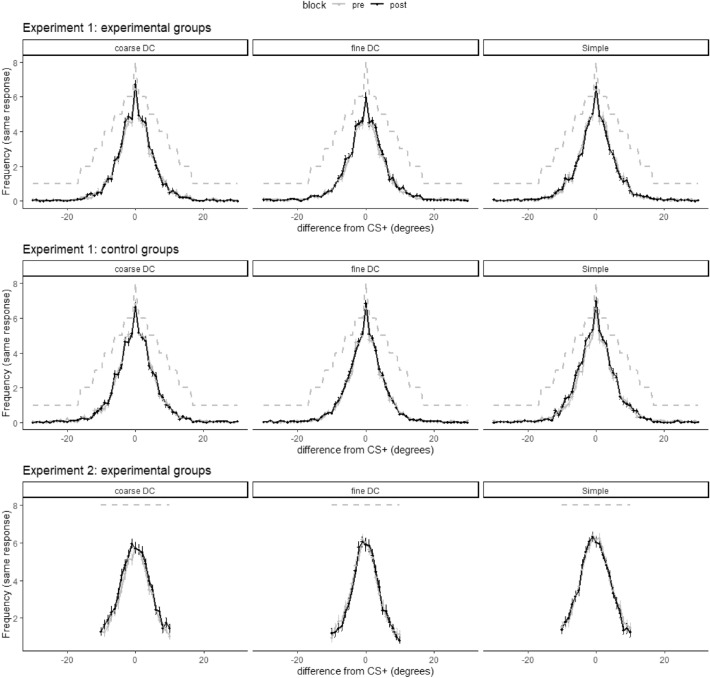
Averaged responses during the 2AFC discrimination test. The grey lines indicate the total amount of presented trials per stimulus per discrimination task. Error bars denote standard errors.

**Figure 6:**
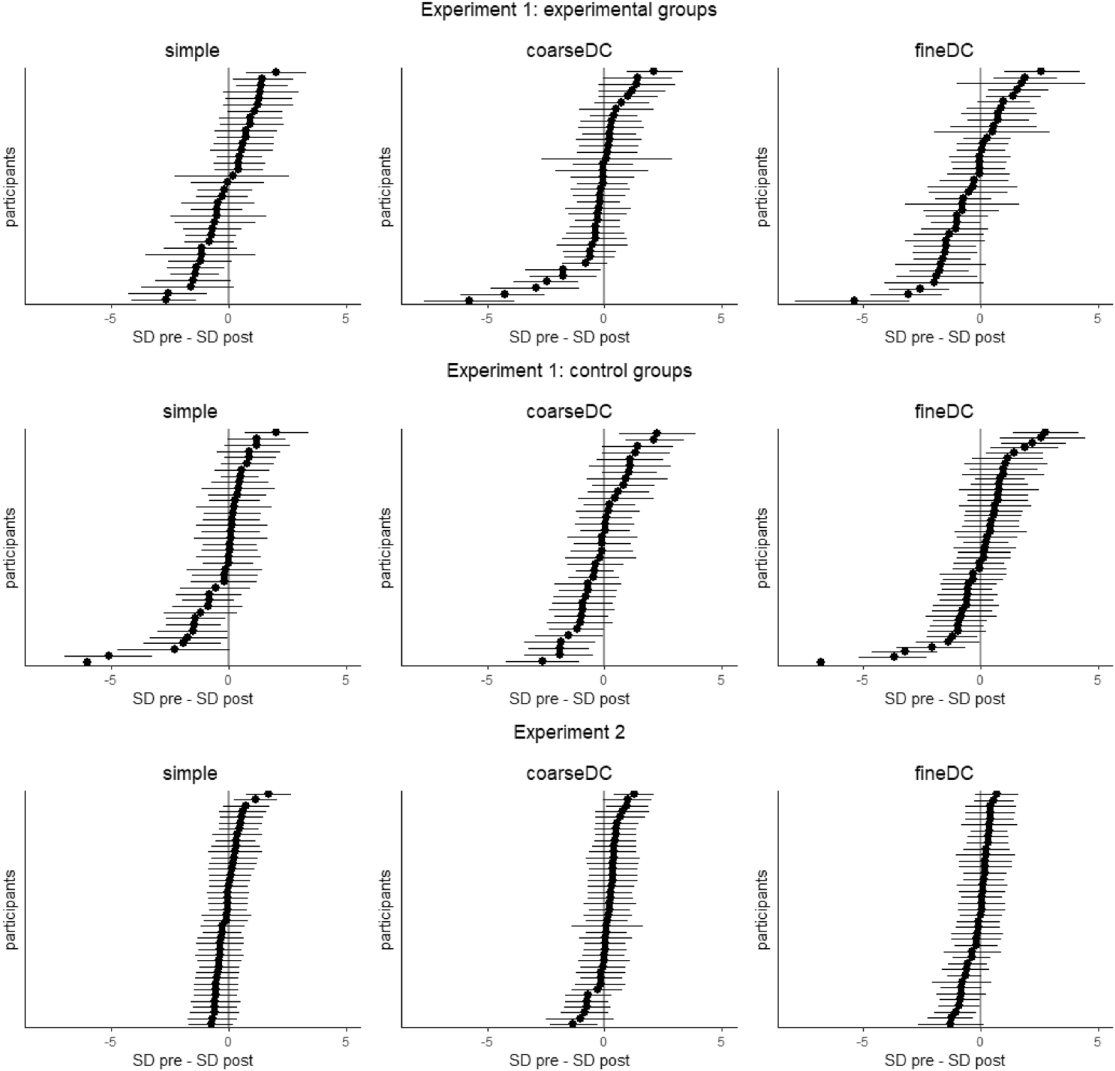
The difference in discrimination acuity between pre- and post-conditioning, as reflected by the individual posterior estimates of the standard deviations of the fitted Normal distributions in the experimental groups from Experiment 1 (top panel), the control groups from Experiment 1 (middle panel), and the experimental groups from Experiment 2 (bottom panel). The dots represent the posterior means. Error bars denote the 95% credible intervals.

As visible in Fig. 5, there was no change in discrimination acuity from pre- to post-learning, as would be reflected by a change in either the mean or the standard deviation of the fitted Normal distributions. For most groups, there was anecdotal or moderate evidence in favor of the Null hypothesis (i.e., no difference between both group distributions) (see Table 2). As a sensitivity analysis, we reran the model, only including participants of Experiment 2 who passed a pre-defined learning criterium (Response probability of predicting US > .5 for the last 20 CS+ trials, in case of a differential group, also a probability of predicting the US < .5 for the last 20 CS− trials) and found very similar patterns. See SI for the posterior means of the group parameters and their 95% credible intervals. Inspection of the individual posterior distributions of the difference scores between pre- and post-conditioning standard deviations revealed that for most participants, zero was within the 95% credible interval, indicating insufficient evidence for improvements or deterioration in discrimination acuity (Fig. 6).

**Table 2:** Bayes factors.

	Bayes factor—Delta Mu	Bayes factor—Delta SD
Experiment 1		
Simple	0.637	0.247
Fine DC	0.441	0.325
Coarse DC	0.450	0.323
Simple—Control	0.734	0.339
Fine DC—Control	0.556	0.220
Coarse DC—Control	0.454	0.258
Experiment 2		
Simple	0.577	0.243
Fine DC	0.309	0.262
Coarse DC	0.311	0.263

## Discussion

In two online experiments, we investigated the impact of fear learning on the ability to discriminate the stimulus predictive of an aversive outcome from other similar physical stimuli. Retrospective US expectancy ratings in both experiments and US predictions during learning in Experiment 2 indicated that overall, participants learned to predict the aversive outcome. Despite successful learning, we failed to find evidence favoring learning-induced changes in perceptual discrimination acuity. On the contrary, in most groups, we found weak to moderate evidence that aversive learning did not alter discrimination acuity. Furthermore, we did not find evidence for a moderating effect of the type of learning experience. Our findings suggest that other (methodological) aspects, beyond those investigated, may be responsible for the previous incongruent findings.

The lack of an effect in any of the three experimental groups in Experiment 1 was unexpected, given that the adopted procedures closely resembled those used by Stegmann et al.^15^, who found an effect, namely an improvement in the discrimination of low-level visual features. Also, the observed gradients in US expectancy ratings, similar to those reported by Stegmann et al.^15^, indicate successful (differential) learning and rule out the possibility that the absence of the effect was due to learning failures. However, the presence of a symmetrical gradient in the fine DC group (in contrast to the expected asymmetrical gradient in the coarse DF group) raised some concerns regarding differential learning in this particular group^31,32^. In the second experiment, we included US predictions during the acquisition phase to foster learning and measure it more directly. The findings from Experiment 2 further support that the absence of an effect of learning on discrimination acuity did not stem from learning failures in the experimental groups. However, as large interindividual differences existed in learning trajectories, we rerun our analysis after only including participants that met a preregistered learning criterium. This did not alter the findings.

One reason for the different findings across studies may lie in the analysis method for the discrimination data. Both studies fitted distributions with their variance as an index of discrimination acuity. In Stegmann et al.^15^, inferential statistics (i.e., rmANOVAs) on the obtained individual parameter estimates were used, with parameters estimated using the maximum likelihood method. Such an approach does not take the uncertainty regarding parameter estimates (i.e., that other parameters values could have yielded similar results) into account during statistical inferences, while the adopted Bayesian framework in our study did so (as a review see^38,39^. To further investigate the potential impact of analytical approaches on the results, we reanalyzed the data from Stegmann et al.^15^ using the same method as in the current study. Similar to our findings, we found Bayes factors indicating weak to moderate evidence for the absence of an effect (see SI for these additional analyses), thereby contradicting the originally reported findings. Due to the wide 95% credible intervals of parameter estimates in Experiment 1, we increased the number of stimulus presentations in the discrimination task in Experiment 2 to ascertain that the parameter uncertainty was not responsible for the observed lack of an effect. As expected, increasing the number of measurement points per stimulus led to more certain parameter estimates of discrimination acuity (i.e., the SDs) reflected by the narrower 95% credible intervals in Experiment 2 compared to Experiment 1. Despite these reductions in parameter uncertainty, the observed effect remained absent.

The numerous methodological differences between the previous studies prevent an exhaustive discussion. Therefore, we will discuss some potential aspects that may explain the incongruent findings. Although, at first glance, none seem capable of explaining all of the findings. For instance, unlike the studies that found discrimination deteriorations after learning, an absolute stimulus identification task has never been adopted in the studies that found learning-induced improvements. During an absolute stimulus identification task, participants must identify the CSs from memory along a range of presented stimuli with large interstimulus intervals. Compared to two-alternative-forced-choice (or 3-AFC) tasks with brief or no stimulus intervals, this task depends more on memory processes. However, Shalev et al.^13^ demonstrated reduced stimulus discrimination using both a two-alternative forced choice task and an adjusted staircase method (see also^11^, rendering this explanation unlikely. Another cause of the divergent findings may be the modality of the CS. Most studies that reported improvements in discrimination acuity used odors as CSs, while auditory CSs were used in most studies that found deteriorated discrimination. Yet, Rhodes et al.^14^ found discrimination improvements using Gabor patches and Shalev et al.^13^ demonstrated learning-induced deteriorations using both auditory and different visual CSs. Finally, we used visual USs, which may be less aversive than some of the other used US modalities in previous human research (somatosensory, auditory, olfactory) and are certainly less aversive than the electrical stimulations that are usually employed in animal research^5^. Our different US pictures were perceived as unpleasant but only mildly arousing (see SI). Given that learning-induced discrimination changes have also been observed for positive USs (e.g., monetary reward), it could be that not the valence but the degree of arousal may be an essential determinant to observe the effect. However, as arousal ratings were not reported for most of the previously used USs, it remains unclear whether aversive shocks, loud noises, negative sounds, monetary loss, or aversive odors were more arousing than the aversive pictures used in our study.

Of note, the online format of the current study meant reduced control over external factors such as the size, luminance, and quality of the display that may affect discrimination acuity. These factors may have not only influenced discrimination performance but also may moderate differential learning, as stimuli that have more contrast and a larger visual angle are easier to distinguish. However, we argue that technical differences—being a constant property within participants—are canceled out in the pre-post comparisons, which were the primary outcome. Another less controllable factor in online research compared to in the laboratory is the presence of distractors. However, several inclusion criteria were implemented to ensure that only attentive participants were included (i.e., US memory score < 7, amount of missing data > 15%, and the question about serious responding). Furthermore, a re-analysis of the data with only participants who demonstrate learning did not yield any different conclusions, rendering the possibility that our findings stem from inattentive responding rather unlikely. A final limitation is that the exclusion criteria for the memory task only applied to the experimental but not to the control groups. However, since the number of participants excluded due to bad memory performance was relatively small (21 of 449 participants that completed the studies), we assume this did not lead to systematic bias between groups. Apart from the inherent limitations of an online study, there are also several advantages to online research. For instance, it allows for a very conservative power analysis with a large sample size. Given the ease of data collection, we imposed stricter inclusion criteria compared to most laboratory studies, especially regarding participants’ behavior during the experiment. Another advantage is that it enabled us to obtain a more heterogenous and representative sample with participants from various countries and ethnical backgrounds, which should increase the generalizability of our findings (see SI for sample demographics)^40^. Finally, past research has validated experimental online research to study various cognitive and affective processes^41,42^.

In conclusion, this study further contributes to the ongoing debate if associative learning can modify how we perceive our surroundings and, if so, to what extent. Contrary to prior assumptions, our findings indicate that aversive learning does not necessarily enhance perceptual discrimination, nor does the presence of additional safety cues appears to affect this outcome. Overall, our findings and the inconsistent results of previous research suggest that conclusions regarding any modulatory effects of associative learning on perception should be made cautiously. Especially as re-analysis of some previously reported positive findings actually revealed weak to moderate evidence in favor of the absence of an effect.

### Supplementary Information

Supplementary Information.

## Data Availability

We preregistered the studies on the Open Science Framework (https://osf.io/hz2sa; https://osf.io/7wn3g) and explicitly mention any deviations. The datasets generated and/or analyzed during the current study are available in the Open Science Framework repository, https://osf.io/rkbp9, together with all analyses code and the experiment.
