# Supplementary material for: Examining the impact of cue similarity and fear learning on perceptual tuning
Source: Sci Rep. 2023 Aug 10;13:13009. doi: 10.1038/s41598-023-40166-w (PMC10415342; doi:10.1038/s41598-023-40166-w)
Supplement: Supplementary file 1 — Supplementary Information. [file 41598_2023_40166_MOESM1_ESM.docx]

Supplemental information of

**Examining the impact of cue similarity and fear learning on perceptual tuning.**

Authors:

Jonas Zaman, Kenny Yu, Marta Andreatta, Matthias J Wieser, and Yannik Stegmann

**1. Participants**

The animal study of Aizenberg & Geffen (2013), which this study aims to replicate in humans, found a large effect size (f = 1.84). Another recent study in humans (Stegmann et al. 2021) on which this study builds further reported an effect of f = .386 (based on the partial eta squared from the learning type * phase interaction). For our purpose, a re-analysis of that data with only the groups of interest (called Diff+ and Gen+) yielded f = .360. The sample size calculation was done using G*Power 3.1.9.7 for a mixed ANOVA, with f = .15, alpha = .05, Power = 0.95, Groups = 6, Measures = 2, Corr = 0.5, Sphericity = 1. For Experiment 2, the following setting were used for the power sample size calculation: a mixed ANOVA, with f = 0,184, alpha = .05, Power = 0.95, Groups = 3, Measures = 2, Corr = 0.5, Sphericity = 1.

***Table S1. Overview of the participants before and after exclusions.***

|  | started | completed | After exclusions |
| --- | --- | --- | --- |
| Experiment 1 | 323 | 278 | 245 |
| Experiment 2 | 187 | 172 | 121 |

**Demographics**

In experiment 1, 20 of the participants that completed the study were excluded due to bad performance in the memory task. For the final sample, the mean age was 31.07 years (*sd* = 10.85). 57% were males and 41% females, the rest were either non-binary, transgender or did not want to disclose their gender. 86 % of the sample was right-handed. Most participants came from Italy (n = 55), Germany (n = 46), UK (n = 72), South Africa (n = 16) (other countries n < 15).

In experiment 2, only 2 participants did not meet the criteria for the memory task. The mean age for the final sample was 37.14 years (*sd* = 15.28). 55% were males and 41% females, the rest were either non-binary, or did not want to disclose their gender. 86 % of the sample was right-handed. Most participants came from the UK (n = 68), South Africa (n = 40), USA (N = 24) and Poland (17) (other countries n < 15).

**2. US arousal and valence ratings**


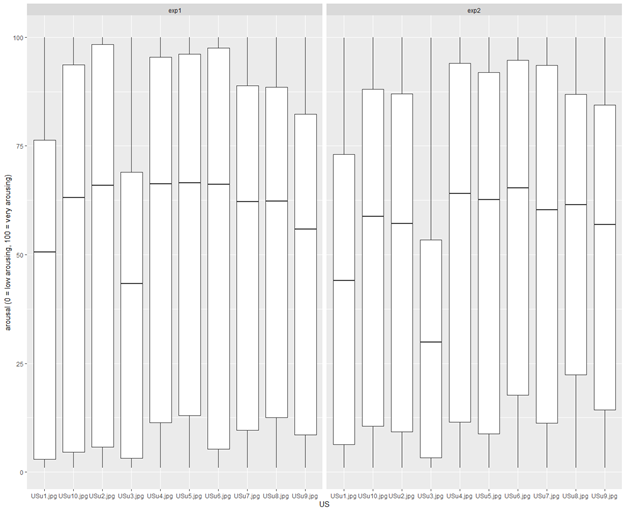


Figure S1. Boxplots per US of arousal ratings.


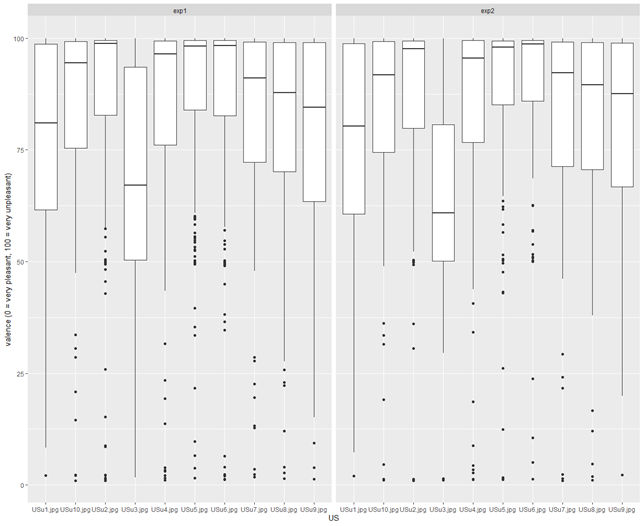


Figure S2. Boxplots per US of valence ratings.

**3. US expectancy**

Table S2. Omnibus Type III tests on the fixed effects using the Satterthwaite's method

|  | ***Df's*** | ***F*** | ***p*** |
| --- | --- | --- | --- |
| **Experiment 1** |  |  |  |
| Orientation | 8, 1912 | 17.43 | < .001 |
| Condition | 2, 239 | 0.95 | .340 |
| US | 1, 239 | 20.77 | <.001 |
| Orientation × Condition | 16, 1912 | 2.74 | < .001 |
| Orientation × US | 8, 1912 | 16.39 | < .001 |
| Condition × US | 2, 239 | 2.12 | .123 |
| Orientation × US × Condition | 16, 1912 | 3.77 | < .001 |
| **Experiment 2** |  |  |  |
| Orientation | 8, 944 | 28,48 | < .001 |
| Condition | 2, 118 | 20.15 | < .001 |
| Condition × Orientation | 16, 944 | 7.33 | < .001 |

**4. Threat ratings**

Threat ratings were analyzed with an identical rmANOVA for the pre-learning and the post learning threat ratings, separately, identical to (Stegmann et al., 2021).

**4.1. Pre-learning**

Unexpectedly we found already differences between orientation pre-learning in threat ratings [stimulus: *F*(8,1912) = 4.45, *p* < .001, *η^2^_p_ =* .018]. All other main and interaction effects were not significant [US: *F*(1,239) = 0.002, *p* = .96, *η^2^_p_ =* .001; Condition: *F*(2,239) = 1.16, *p* = .32, *η^2^_p_ =* .01; US × Condition: *F*(2,239) = 0.41, *p* = .61, , *η^2^_p_ =* .003; US × Stimulus: *F*(8,1912) = 090, *p* = .52, *η^2^_p_ =* .004; Condition × Stimulus: *F*(16,1912) = 1.08, *p* = .37, *η^2^_p_ =* .009, US × Condition× Stimulus: *F*(16,1912) = 0.73, *p* = .77, *η^2^_p_ =* .006] (see Fig. S3).


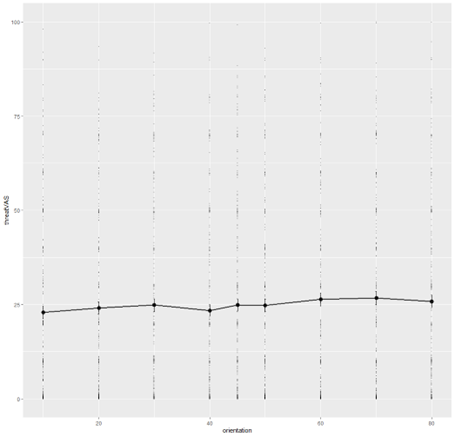


Figure S3. Average and individual pre-learning threat ratings. Errors bars denote standard errors.

**4.2. Post-learning**

There was a main effect of US [*F*(1,239) = 5.68, *p* = .018, *η^2^_p_* = .023], Condition [*F*(2,239) = 3.85, *p* = .023, *η^2^_p_* = .031], Stimulus [*F*(8,1912) = 9.25, *p* < .001, *η^2^_p_* = .037], no interaction between US and condition [*F*(2,239) = 1.79, *p* = .17, *η^2^_p_* = .015], a interaction between condition and stimulus [*F*(16,1912) = 3.03 *p* < .001, *η^2^_p_ =* .025], US and stimulus [*F*(8,1912) = 9.35, *p* < .001, *η^2^_p_ =* .038] and a significant three-way interaction [*F*(16,1912) = 3.11 *p* < .001, *η^2^_p_ =* .025]. Follow-up analysis: Threat ratings did not differ across the stimulus range between the three control groups (US = no.US: Stimulus × Condition: *F*(2,123) = .14, *p* = .32, *η^2^_p_ =* .003), while they differed between the experimental conditions (US = US: Stimulus × Condition: *F*(10.24, 593) = 3.87, *p* < .001, *η^2^_p_ =* .033). Additionally we tested for a difference in threat ratings between the CS+ and CS- in both differential groups. Ratings were significantly lower for the CS- compared to the CS+ in the coarseDC group [45° vs 10°: *t*_(39)_ = 4.80, *p_cor_* < .001] but not in the fineDC group [45° vs. 40°*t*_(39)_ = 0.54, *p_cor_* = .59]. Neither in the coarseDF and SC groups lower ratings were found for the 40° gabor patch compared to 45° (CS+) [*t*_(39)_ = 1.78, *p_cor_* = .083; *t*_(38)_ = 1.69, *p_cor_* = .099](Fig. S4).


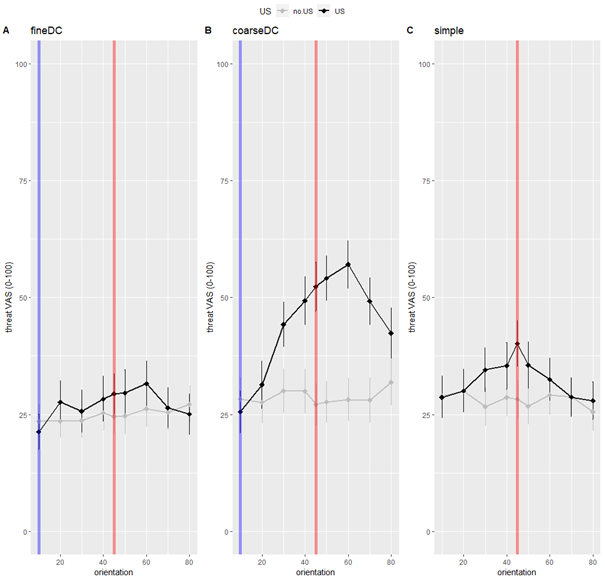


Figure S4. Average and individual post-learning threat ratings. Errors bars denote standard errors.

**5. Posterior mean group parameters and 95% credible intervals**


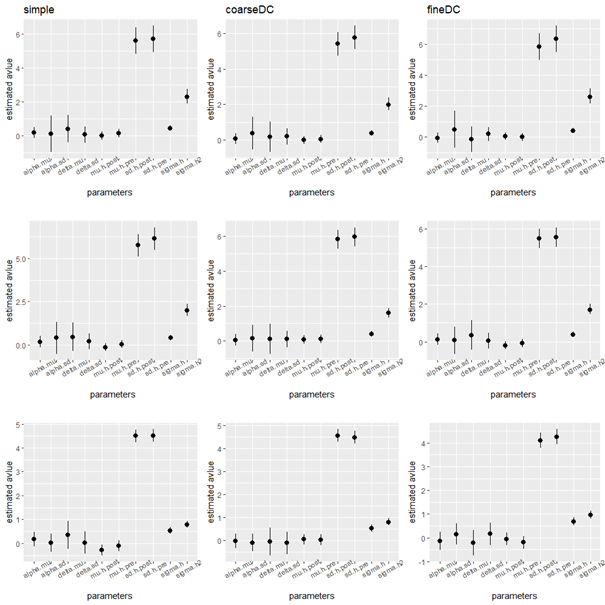


*Figure S5. Posterior means for the different group parameters. Top: the experimental groups from Experiment 1, Middle: control groups of Experiment 1. Bottom: experimental groups of Experiment 2. The dots are posterior means and the error bars denote the 95% credible intervals.*

**6. Local asymmetry effects**

Since the reported Bayes factors provided weak to moderate evidence for the absence of any change in discrimination acuity, we decided not to conduct the preregistered follow-up analysis that focuses on potential asymmetry effects in changes in discrimination acuity across the entire test dimension (by comparing changes on the CS- side to changes on the non-CS- side). However, a second preregistered follow-up that focused on more local asymmetry effects was performed in order to capture more subtle effects. This was done by comparing the summed amount of 'same' responses during the discrimination task for stimuli that maximally differed 5° in orientation to the CS+ (excluding the CS+). For Experiment 1, the linear mixed model comprised of the within-subjects predictors’ Side (CS- side or non-CS- side) and Block (pre and post). Condition (categorical predictor, simple, fine, coarse) and US (categorical predictor, US or no US) were the between-subjects predictors plus all potential interactions. A subject-dependent intercept was included. For Experiment 2, the predictor US was omitted from the model as there were no control groups.

**6.1. Experiment 1**

We found a significant interaction between Block , Condition and US [*F*(2,3462) = 10.29, *p* < .001] as well as a main effect of Block and US [Block: *F*(1,3462) = 8.22, *p* = .004, US: *F*(1,226) = 5.14, *p* = .024]. Follow-up contrast on the model's estimated means revealed no change in the control groups (*p*'s> .20) apart from a response increase in the simple group (*p* = .0008) from pre- to post-conditioning. In the experimental groups, the patterns differed with no change in the Simple group (*p* = .15) and a significant increase in responding for the differential conditioning groups (*p*'s < .01). All other main and interaction effects were not significant (*p*'s > .18).

**6.2. Experiment 2**

This time we found significant effects of Side [*F*(1,1806) = 9.59, *p* = .002], Block [*F*(1,1806) = 4.25, *p* = .040] and a Side by condition interaction [*F*(2,1806) = 4.68, *p* = .009] with more responding on the non-CSM side for the Simple and fine DC groups (*p* = .017 and *p* < .001) but not the coarse DC group (*p* = .549). All other main and interaction effects were not significant (*p*'s > .11).

**7. Correlation analysis**

We assessed the correlation between discrimination acuity (SDpre, SDpost, and SDpre-SDpost) and differences in generalization gradients. For this purpose, a generalization index (GI) was calculated as follows: individual slope estimates of linear regressions (over the CS+ flipped stimulus space with equidistant stimuli merged) on difference scores of the US expectancy ratings (i.e., stimulus ratings – the CS+ rating of that subject). These correlations were run per group separately. Uncorrected p-values and correlations are reported in table S3. The correlations with STAI-T could not be performed due to a technical error. Reported *p*-values are uncorrected for multiple testing.

**Table S3. Correlations**

|  | **Generalization index** |
| --- | --- |
| **Experiment 1 – Simple (N = 40 )** |  |
| SD_pre_ | *r* = -.05, *p* = .78 |
| SD_post_ | *r* = -.13, *p* = .46 |
| SD_pre_-SD_post_ | *r* = -.15, *p* = .40 |
| **Experiment 1 – Coarse differential (N = 40 )** |  |
| SD_pre_ | ***r* = .34, *p* = .04** |
| SD_post_ | *r* = .26, *p* = .12 |
| SD_pre_-SD_post_ | *r* = -.17, p = .32 |
| **Experiment 1 – Fine differential (N = 39 )** |  |
| SD_pre_ | *r* = .30, *p* = .06 |
| SD_post_ | ***r* = .34, *p* = .04** |
| SD_pre_-SD_post_ | *r* = .01, *p* = .97 |
| **Experiment 2 – Simple (N = 41)** |  |
| SD_pre_ | ***r* = .54, *p* < .001** |
| SD_post_ | ***r* = .48, *p* = .001** |
| SD_pre_-SD_post_ | *r* = -.13, *p* = .41 |
| **Experiment 2 – Coarse differential (N = 41)** |  |
| SD_pre_ | *r* = .14, *p* = .39 |
| SD_post_ | *r* = -.01, *p* = .93 |
| SD_pre_-SD_post_ | *r* = -.21, *p* = .19 |
| **Experiment 2 – Fine differential (N = 39)** |  |
| SD_pre_ | *r* = -.08, *p* = .65 |
| SD_post_ | *r* = -.11, *p* = .52 |
| SD_pre_-SD_post_ | *r* = -.71, *p* = .66 |

8. **Re-analysis of Stegmann et al. (2021)**

Here we reanalyzed the data of Stegmann et al. (2021) using the above described model to investigate whether inferences based on mean parameter estimates and their uncertainty yielded a different conclusion. As visible in Fig. S6, although many mean difference scores are indeed below 0 ine the Diff groups, for many individuals the 95% credible are quite wide including 0 suggesting these difference score do not actually differ from 0.


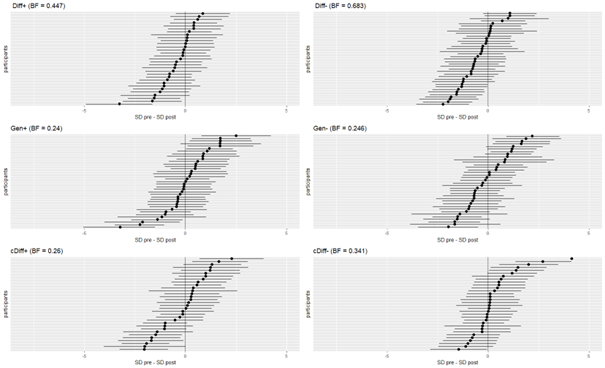


Figure S6. Posterior individual means for the difference scores and their 95% credible intervals. Bayes factor testing the change in SD for pre to post-learning are reported in brackets.
